# Graphene Nanofibers by Integrated Manufacturing of Electrospinning and Laser Graphitization for Miniaturized Energy Storage Devices

**DOI:** 10.1002/advs.202414607

**Published:** 2025-03-31

**Authors:** Bumjun Park, Shirin Movaghgharnezhad, Seung Min Lee, Yonghyeon Park, Sejin Son, Yun Suk Huh, Pilgyu Kang

**Affiliations:** ^1^ Department of Biological Sciences and Bioengineering Nano Bio High‐Tech Materials Research Center Inha University Michuhol‐gu Incheon 22212 Republic of Korea; ^2^ Department of Mechanical Engineering George Mason University Fairfax VA 22030 USA; ^3^ Quantum Science and Engineering Center George Mason University Fairfax VA 22030 USA

**Keywords:** carbon nanofibers, electrospinning, fluorinated polyimide nanofibers, graphene nanofibers, laser graphitization, micro‐supercapacitors

## Abstract

Carbon nanofibers (CNFs) are emerging as promising materials for miniaturized energy storage devices (MESDs) due to their high specific surface area, excellent electrochemical performance, low internal resistance, and durability. Their versatility and tunability make them ideal candidates for various applications, making CNFs a key player in advancing compact and efficient energy storage solutions. Nonetheless, CNFs necessitate an extra step involving either physical or chemical treatments to regulate their morphology, augment surface area, and create micropatterns suitable for MESDs. Here, innovations in material fabrication using an integrated manufacturing process are reported that combines electrospinning and laser‐induced graphitization to create graphene nanofibers (GNFs) from fluorinated polyimide nanofibers (fPI NFs). Initially, electrospinning yields uniformly sized and shaped fluorinated poly(amic) acid nanofibers, which are subsequently thermally imidized to form fPI NFs. Laser photothermal treatment of fPI NFs generates hierarchical meso‐ and nanopores in GNFs, enhancing specific surface area and electrochemical properties, including specific capacitance, cyclic stability, rate capability, areal capacitance, power density, and energy density. This integrated approach synergistically fabricates GNFs for MESD applications, particularly GNF‐based micro‐supercapacitors (MSCs), demonstrating a remarkable areal capacitance and an aerial energy density two orders of magnitude higher than MSCs based on laser‐induced graphene derived from conventional polyimide film.

## Graphical abstract

Integrated manufacturing combines electrospinning and laser graphitization to produce graphene nanofibers (GNFs) from fluorinated polyimide (fPI). GNFs, with meso‐ and micropores, enhance micro‐supercapacitors performance, demonstrating energy density over two orders of magnitude higher than conventional ones, making them ideal for miniaturized energy storage devices.

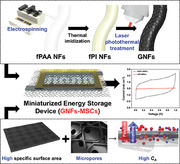

## 1 Introduction

Fiber‐based carbonaceous materials find extensive use in electrochemistry, particularly in supercapacitors, Li‐ion batteries, and electrochemical sensors.^[^ ^1^ ^]^ Their notable attributes, including a high specific surface area and excellent electrical conductivity, make them well‐suited for improving the overall performance and efficiency of these applications. Carbon nanofibers (CNFs) have emerged as a particularly promising material for the advancement of micro‐supercapacitors, owing to their substantial surface area, exceptional electrical conductivity, and robust mechanical strength.^[^ ^2^ ^]^ Various techniques exist for producing CNFs, each offering unique advantages and applications.^[^ ^3^ ^]^ Catalytic chemical vapor deposition decomposes carbon‐containing gas over a metal catalyst at high temperatures for precise control of nanofiber morphology.^[^ ^4^ ^]^ Electrospinning creates charged jets from polymer solutions, solidifying into nanofibers upon solvent evaporation using high voltage.^[^ ^5^ ^]^ Template‐assisted synthesis uses porous templates as scaffolds for controlled nanofiber growth.^[^ ^6^ ^]^ The arc discharge method vaporizes and condenses carbon atoms into nanofibers through high electrical potential in an inert gas environment.^[^ ^7^ ^]^ Chemical vapor deposition on patterned substrates aids controlled growth of CNFs in specific regions.^[^ ^8^ ^]^ Hydrothermal carbonization carbonizes biomass or carbon precursors in a hydrothermal environment, producing CNFs with controlled morphology and surface chemistry in an eco‐friendly manner.^[^ ^9^ ^]^

Despite the availability of CNFs production methods, it is crucial to comprehend the limitations of each technique, particularly when applied to micro‐supercapacitors.^[^ ^10^ ^]^ Controlling the nanofiber structure presents challenges that greatly influence the overall performance of micro‐supercapacitors. Research emphasizes the significance of maintaining a balanced micropore/mesopore ratio in carbon fiber structures to achieve high‐rate capacitive performance.^[^ ^11^ ^]^ Challenges in scalability, cost, energy efficiency, process control, uniformity, and reproducibility further complicate the seamless integration of CNFs into micro‐supercapacitors.^[^ ^12^ ^]^ Environmental concerns tied to specific production techniques also underscore the necessity for eco‐friendly and sustainable manufacturing processes.^[^ ^13^ ^]^ Additionally, integrating CNFs may pose compatibility issues with other components like electrodes and electrolytes, potentially compromising device efficiency or causing failure.^[^ ^14^ ^]^

The laser‐induced graphitization method offers significant advantages in supercapacitors and micro‐supercapacitors through cost‐effective manufacturing process with laser technology, producing customized porous graphene structures.^[^ ^15^ ^]^ This technique enables precise carbon micropatterning in a one‐step process at room temperature, positioning it as an efficient option for large‐scale energy storage device production. However, achieving an optimal micropore/mesopore ratio with polymer‐based films remains a challenge, limiting their potential to enhance the energy storage density of micro‐supercapacitors. Electrospun nanofibers, generated through electrospinning, are versatile materials with notable surface area‐to‐volume ratios, exceptional mechanical properties, and customizable porosity.^[^ ^16^ ^]^ These nanofibers play a crucial role in micro‐supercapacitors by facilitating effective electrolyte transport and offering improved ion accessibility, contributing to superior energy storage capabilities.^[^ ^17^ ^]^ Their robustness ensures long‐term stability during charge and discharge cycles. The incorporation of graphene into electrospun nanofibers further enhances performance, increasing charge transfer efficiency, specific capacitance, and energy storage capacity.^[^ ^18^ ^]^ This integration presents an opportunity to advance energy storage technologies and create high‐performing micro‐supercapacitors for diverse applications.

The synthesis of CNFs through electrospinning has emerged as a promising technology in the field of capacitive energy storage, offering excellent performance due to their high electrical conductivity and specific surface area. High‐quality CNFs are typically synthesized via heat treatment processes, including oxidation and carbonization using Polyacrylonitrile (PAN) as a precursor.^[^ ^19^ ^]^ PAN‐based CNFs enable fast ion transport and exhibit high energy density, making them ideal supercapacitor electrode materials due to their superior electrical conductivity and structural stability. Additionally, eco‐friendly CNFs can be fabricated using bio‐based precursors, such as lignin, which are cost‐effective, environmentally friendly, and well‐suited for energy storage applications.^[^ ^20^ ^]^ Lignin‐based CNFs provide high specific surface area and electrical conductivity, enhancing both double‐layer capacitance and pseudo‐capacitance. However, conventional CNFs face challenges, such as difficulty in controlling pore size due to the complexity of optimizing porous structures and the need for high temperatures and prolonged durations during the carbonization process.^[^ ^21^ ^]^ Therefore, the development of advanced graphitization techniques for nanofibers is essential to address these limitations and improve their performance.

**Figure 1:**
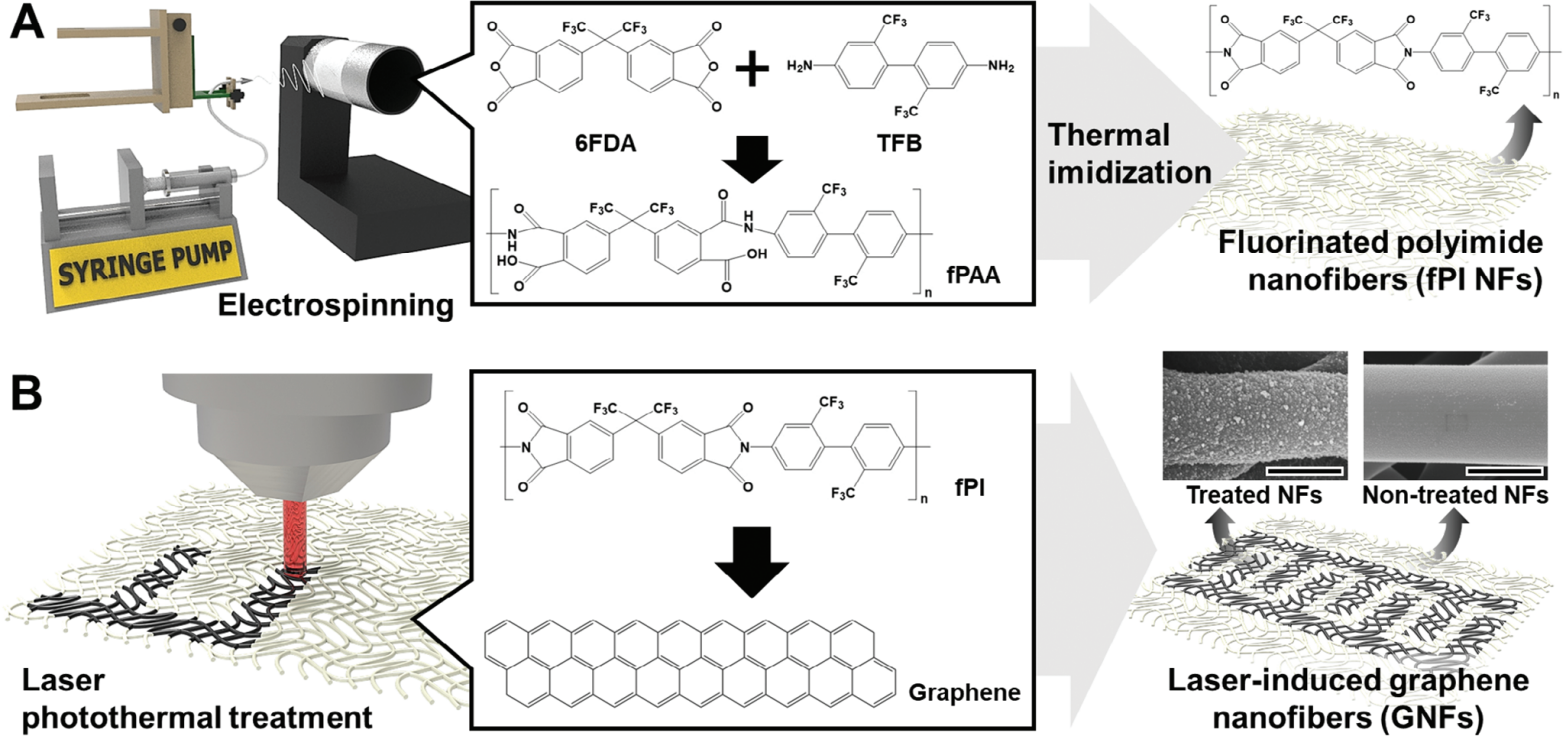
Integrated manufacturing of graphene nanofibers (GNFs) using electrospinning and laser photothermal processing. A) Electrospinning fPAA NFs to synthesize fPI NFs through thermal imidization at 250.0 °C for 2 h. B) laser photothermal treatment for GNF generation, transforming amorphous carbons in fPI NFs into crystalline graphitic structures.

We introduce an integrated manufacturing method for graphene nanofibers (GNFs) through a combination of electrospinning and laser graphitization of fluorinated polyimide nanofibers (fPI NFs) (**Figure** **1**). Initially, electrospun fPI NFs are created, allowing precise porosity modulation and significant surface area augmentation in micro‐supercapacitors (MSCs). The incorporation of these electrospun separators into miniaturized energy storage devices (MESDs) holds promise for optimizing energy storage performance through controlled porosity and increased surface area. Subsequently, the fPI NFs undergo laser photothermal treatment, modifying the structure and properties through transitions from sp^3^ carbon to sp^2^ carbon and vaporization of fluorine. Compared to polyimide films, electrospun fPI NFs enhance the laser graphitization process with structural controllability and higher thermal strength. Laser graphitization of fPI NFs enables the vaporization of fluorine, creating desirable meso‐ and micro‐scale porous structures and increasing specific surface area. The resulting GNFs serve as functional materials for GNFs‐based micro‐supercapacitors (GNFs‐MSCs), demonstrating a high areal capacitance of 16.8 mF cm^‒2^, over an order of magnitude higher than that reported electrical double‐layer capacitance (EDLC)‐based MSCs. Additionally, the areal energy density of GNFs‐MSCs is nearly two orders of magnitude higher than that of laser‐induced polyimide film‐based micro‐supercapacitors (LIPI‐MSCs). GNFs with tunable microstructural control, an order‐of‐magnitude high specific surface area, and enhanced electrochemical performance, show great potential as highly efficient next‐generation MESDs.

## 2 Results and Discussion

### 2.1 Integrated Electrospinning and Laser Photothermal Manufacturing for Microporous‐Structured GNFs

To synthesize microporous‐structured GNFs, we employed a combination of electrospinning and laser photothermal manufacturing (Figure 1). The three‐step process involved electrospinning fluorinated poly(amic)acid nanofibers (fPAA NFs), followed by the synthesis of fluorinated polyimide nanofibers (fPI NFs), and laser photothermal processing of fPI NFs. Initially, fPI NFs were produced through electrospinning fPAA NFs, followed by thermal imidization at 250.0 °C for 2 h (Figure 1A). Electrospinning is a continuous manufacturing method for fibers of nanometers to micrometers in diameter by applying high voltage to a polymer solution.^[^ ^22^ ^]^ This process ensures nanofibers with high uniformity, diameter consistency, and high specific surface area, enhancing material properties and functional performance across various applications.^[^ ^23^ ^]^

**Figure 2:**
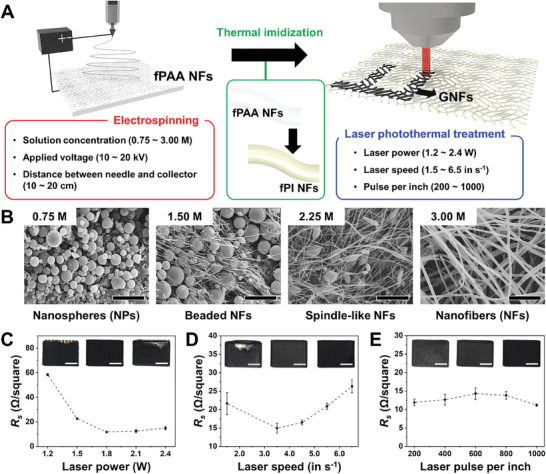
Optimal GNFs fabrication through parameter control of electrospinning and laser photothermal treatment. A) Schematic illustration showing parameters of electrospinning and laser photothermal treatment to optimize uniform GNFs fabrication. B) SEM images of electrospun fPAA NPs or NFs under various solution concentrations with scale bars indicating 10.0 µm. Sheet resistance (*R_s_*) values of fabricated GNFs controlling various laser parameters such as C) power, D) speed, and E. PPI with optical images of NFs (insets). All scale bars of inset images indicate 1.0 mm.

Concentration of solution, applied voltage, and distance between needle and collector (DNC) are the factors to consider uniform fPAA NFs fabrication (**Figure** **2**A). Especially, concentration of fluorinated poly(amic) acid (fPAA) solution, comprising 2,2′‐bis(trifluoromethyl)benzidine (TFB) and 4,4′‐(hexafluoroisopropylidene)diphthalic (6FDA) dissolved under N,N‐dimethylacetamide (DMA), played a crucial role in achieving uniform size and shape of fPI NFs during electrospinning, significantly influencing the resulting nanofiber properties. To investigate the impact of concentration and viscosity on uniformity, the concentration of fPAA solutions was varied to four different states, each of which can produce different morphology of fPI NFs (Figure 2B). The low concentration (0.75 m solution) led to the formation of fluorinated polyimide nanoparticles (fPI NPs) due to high surface tension.^[^ ^24^ ^]^ Higher concentrations produced beaded nanofibers, including spherical beads (1.50 m solution) and spindle‐like beads (2.25 m solution).^[^ ^25^ ^]^ The optimal concentration of 3.00 m yielded smooth and uniform fPAA NFs. At concentrations beyond 3.00 m, excessive viscosity prevented smooth solution release, compromised nanofiber uniformity, and led to the formation of amorphous structures rather than well‐defined nanofibers. When the applied voltage and DNC were changed at a solution concentration of 3.00 m, it was confirmed that there was no significant effect on the structure of the nanofibers (Figure S1, Supporting Information). Subsequently, optimized fPAA NFs were converted into fPI NFs through imidization and annealing at 250 and 300 °C, respectively. The conversion process involved various physical and chemical transformations, resulting in the formation of fPI nanofibers.^[^ ^26^ ^]^ Therefore, the 3.00 m fPAA solution, ensuring uniform size and shape, was chosen for subsequent laser graphitization.

GNFs were synthesized using a laser photothermal process applied to fPI NFs as illustrated in Figure 1B. Unlike traditional methods like Chemical Vapor Deposition (CVD), laser graphitization proved faster and simpler, facilitating rapid patterning and localized graphitization. The incident light's intensity, tailored to the laser‐treated material, dictated the local temperature via operation variables. Employing a 10.6 µm CO_2_ laser, the focused area surpassed 2500 °C, disrupting covalent bonds in fPI NFs and releasing H_2_, N_2_, O_2,_ and F_2_ gases.^[^ ^15a^ ^]^ The high temperature induced the evaporation of the gaseous molecules, resulting in multi‐scale meso‐ and micropores within the fibrous structure of graphene.^[^ ^27^ ^]^ Notably, the release of gaseous fluorine played a crucial role in generating hierarchical nano‐ and microstructures.^[^ ^28^ ^]^ During the laser processing, the yield ratio was calculated as 88.86%, indicating that a portion of nanofibers was vaporized or lost due to thermal decomposition. The laser parameters (power, speed, and pulses per inch) were optimized using fPI NFs with 3.00 m fPAA, resulting in GNFs with superior electrical conductivity (Figure 2A). Varying laser power (1.2 to 2.4 W) while keeping the laser speed constant (3.5 s^−1^) and maintaining 1000 PPI, 1.8 W yielded optimal conductivity (Figure 2C; Figures S2A, S3A, Supporting Information). Lower powers graphitize fPI NFs and higher powers cause graphene flake cracks. Laser speed directly affects the uniformity of graphitization and the conductivity of the nanofibers. Excessively slow speeds increase local thermal damage and structural defects due to excessive energy transfer, while excessively fast speeds result in incomplete graphitization. Within the optimal velocity range of 1.5 to 6.5 in s^‒1^, energy is uniformly transferred, enabling the formation of a well‐graphitized structure and ensuring high conductivity. Specifically, at a laser speed of 3.5 in s^‒1^, the sample exhibited the lowest sheet resistance (*R_s_*) value (Figure 2D; Figures S2B, S3B, Supporting Information), while variations in PPI (200 to 1000) had negligible impact on conductivity (Figure 2E; Figures S2C, S3C, Supporting Information). Both *R_s_* and I‐V measurements consistently identified the optimal laser conditions for GNF production as 1.8 W, 3.5 in s^‒1^, and 1000 PPI.

### 2.2 Characterization of Laser‐Induced Graphene Nanofibers

**Figure 3:**
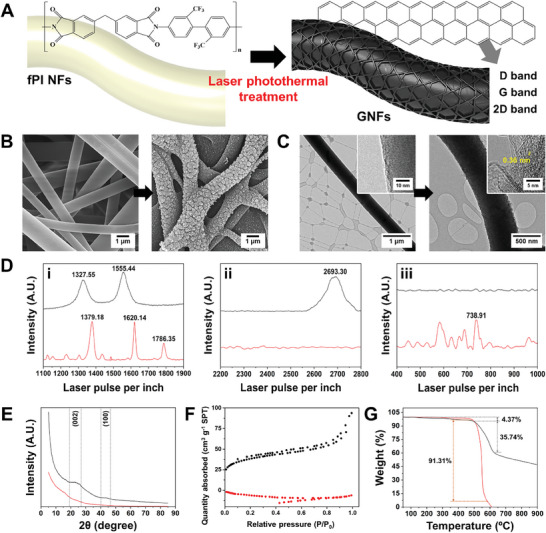
Morphological and spectroscopic characterizations of fPI NFs and GNFs. A) Schematic illustration showing the modification of chemical structure after laser photothermal treatment. B) SEM images and C) TEM images before and after laser photothermal treatment. D) Raman spectra of fPI NFs and GNFs under range of i) 1100 to 1900 cm^−1^, ii) 2200 to 2800 cm^−1^, and iii) 400 to 1000 cm^−1^. E) XRD spectra, F) BET analysis, and G) thermogravimetric analysis (TGA) of fabricated NFs. The black and red curves indicate GNFs and fPI NFs, respectively.

Specific characterization and comparison of fabricated nanofibers proceeded to confirm the uniformly formed GNFs using the combination of electrospinning and laser photothermal treatment (**Figure** **3**A). Using optimized laser conditions, we initially examined nanofiber surfaces for morphological and chemical changes, evaluating morphology through scanning electron microscopy (SEM) and atomic lattice using transmission electron microscopy (TEM). Electrospun fPI NFs displayed a smooth surface with an average fiber diameter of 948.18 nm, while GNFs exhibited a rough surface with an average diameter of 930.80 nm and a lattice spacing of 0.36 nm, indicative of graphene lattice. (Figure 3B,C; Figure S4, Supporting Information).^[^ ^29^ ^]^ Graphene flakes on GNF surface indicated substantial meso‐ and micro‐porosity, attributed to laser‐induced photon energy absorption breaking carbon atom bonds and causing gaseous molecule evaporation (O_2_, N_2_, H_2_, and F_2_). EDS analysis confirmed carbon ratio increase (45.07% to 58.74%) and fluorine ratio decrease (36.92% to 21.82%) due to the release of fluorine‐containing groups during laser treatment (Figure S5, Supporting Information).^[^ ^27^ ^]^ Exceeding the optimized laser power caused fPI NFs to melt, resulting in surface contraction and collapsed graphene sheets (Figure S6, Supporting Information). Optimized laser conditions induced significant morphological and chemical changes, including graphitic structure development, graphene flake formation, and shifts in carbon and fluorine ratios. While flexibility cannot be directly evaluated, the structural properties, including sp^2^ carbon networks and hierarchical porosity, suggested high flexibility potential. Additionally, the formation of sphere‐like particles in GNFs was attributed to surface energy minimization, volatile gas release, and local thermal stress during laser photothermal treatment, which promoted structural rearrangement and enhanced electrochemical activity while maintaining hierarchical porosity. This demonstrates the potential for precise structural and morphological adjustments, enabling tailored nanofiber properties, surface modifications, and composite structures for various applications.

To ascertain the optimal laser graphitization parameters for transforming amorphous carbon in fPI NFs into graphitic structures, we conducted a Raman spectroscopy analysis to assess the quality and chemical characteristics of the resulting GNFs. The D, G, and 2D peaks in the Raman spectra confirm the formation of graphite nano‐crystallite GNFs through laser graphitization. (Figure 3D; Table S1, Supporting Information). The D peak originates from in‐plane vibrational modes at the surface of sp^2^ carbon domains, activated by disruptions in sp^2^ bonding, while the G peak corresponds to in‐plane stretching vibration of sp^2^ hybridized carbon atoms within the hexagonal lattice structure.^[^ ^30^ ^]^ With increasing laser power, the D peak intensity initially increases slightly due to localized disruptions but subsequently decreases as defects are annealed, while the G peak intensity steadily increases, indicating enhanced graphitic order. The results are further quantified by the D‐to‐G peak intensity ratio (I_D_/I_G_), which decreases from 1.024 at 1.5 W to 0.761 at 2.1 W (Figure S7A; Table S2, Supporting Information). The results suggest that moderate laser power promotes the reduction of structural defects and the dominance of in‐plane stretching vibrations within sp^2^‐bonded carbons (π‐bonded C═C networks). However, excessive laser power could lead to structural damage, as indicated by deviations in the linear progression of I_D_/I_G_.^[^ ^31^ ^]^ Interestingly, a slight shift in the D and G peaks in the Raman spectrum was observed, likely induced by the unique defect density of samples, chemical environment, and laser processing conditions.^[^ ^32^ ^]^ The 2D peak in GNFs, revealing layer information through a double resonance enhanced two‐phonon lateral vibrational process, exhibited a slight decrease in both the full width at half‐maximum (FWHM) and the 2D‐to‐G peak intensity ratio (I_2D_/I_G_ ratio) with increasing laser power (Table S3, Supporting Information). The diminished FWHM of the 2D peak suggests a well‐ordered single graphene layer on GNF's surface.^[^ ^33^ ^]^ The I_2D_/I_G_ ratio ranging from 1.0 to 0.7 indicates single‐layer graphene synthesis during laser photothermal treatment of fPI NFs.^[^ ^34^ ^]^ Additionally, the disappearance of the C─F vibration peak in GNFs indicates fluorine vaporization, confirming the formation of meso‐ and microporous nanofibers. This Raman spectroscopy analysis affirms the successful conversion of amorphous carbon in fPI NFs into high‐quality single‐layer graphene structures through laser graphitization. This is supported by the absence of the C─F vibration peak and the I_2D_/I_G_ ratio indicating single‐layer graphene synthesis.

X‐ray diffraction (XRD) analysis was performed to assess the crystallinity and structural characteristics of GNFs synthesized through laser photothermal treatment under different laser powers (Figure 3E; Figure S8A, Supporting Information). XRD patterns of GNFs exhibited distinct peaks, including a (002) peak at ≈21.6° and a (100) peak at 43.4°, The (002) peak corresponds to an interlayer spacing (*d*‐spacing) of ≈0.34 nm, indicative of a high degree of graphitization and consistent interlayer stacking, suggesting intact π‐π interactions without significant flaking or separation of graphene layers.^[^ ^35^, ^36^ ^]^ The (100) peak is associated with the hexagonal lattice structure of graphitic carbon, and its increasing sharpness and intensity at higher laser powers reflect enhanced in‐plane crystallinity resulting from laser‐induced structural transformations.^[^ ^37^ ^]^ However, at excessively high laser power (2.1 W) slight broadening of the peaks was observed, which could be attributed to local defects or thermal stress introduced during the process. The consistent *d*‐spacing and the sharp XRD peaks across most laser power levels further confirm the presence of multilayer graphene domains interspersed with a few monolayered sheets. Regarding the intensity below 10°, the observed peak was attributed to Bremsstrahlung effect rather than intrinsic crystallographic properties of fPI NFs and GNFs. The effect, caused by background radiation from high‐speed electron deceleration, combined with scattering signals from substances and residual amorphous components, exaggerates intensity in the specific region. These findings underscore the robustness and effectiveness of the laser graphitization process in producing GNFs with high graphitization, superior crystallinity, and intact structural integrity. Such structural properties highlight the suitability of GNFs for high‐performance electrochemical applications.

Brunauer‐Emmett‐Teller (BET) analysis was further conducted to analyze the surface area and porosity of GNFs and fPI NFs, revealing significant differences in the adsorption characteristics (Figure 3F). GNFs exhibited a BET surface area of 134.1074 m^2^ g^−1^, indicating a high degree of porosity and an extensive surface area suitable for applications requiring surface interactions. Conversely, fPI NFs showed lower nitrogen adsorption, suggesting that laser photothermal treatment effectively increased the surface area, transforming into GNFs with enhanced porosity and adsorption capacity. The BET data for graphene nanofibers (GNFs) exhibit a monotonically decreasing pore size distribution, with a dominant presence of mesopores (2–50 nm) that gradually taper off at larger pore sizes (Figure S8B, Supporting Information). The mesopores are likely attributed to the interconnected structure and alignment of the nanofibers, while micropores (<2 nm) are also present, generated by localized thermal effects and rapid vaporization during the laser photothermal process. The absence of pronounced peaks in the macropore range (>50 nm) suggests that the larger voids between aggregated fibers are relatively less prevalent. This gradual decline in pore distribution highlights the uniformity and hierarchical porosity of the GNFs. Therefore, the enhancement highlighted the role of laser photothermal treatment in improving the porous structure of GNFs, which was highly suitable for surface activity‐dependent applications.

We assessed the molecular and chemical compositions of GNFs using Fourier transform infrared (FT‐IR) and X‐ray photoelectron spectroscopy (XPS). FT‐IR spectra revealed the disappearance of the C─H bond bending in GNFs and CF_3_ bond vaporization in fPI NFs, evidenced by weakened C─F stretching vibrations (Figure S9A; Table S4, Supporting Information). Increasing laser power led to pronounced CF_3_ bond vaporization, demonstrated by the disappearance of the C─F stretching peak at 2.1 W (Figure S9B, Supporting Information). This suggests that higher laser power results in increased fluorine vaporization, leading to higher levels of meso‐ and microporous nanofibers. Moreover, XPS analysis of GNFs produced under optimal laser settings showed an increase in the carbon atomic ratio from 63.02% to 74.46% and a decrease in fluorine from 25.48% to 15.88% after laser treatment (Figure S10 and Table S5, Supporting Information). The content of sp^2^ carbon increased from 12.05% to 25.62%, while CF_3_ content decreased from 19.51% to 10.29%, indicating the formation of sp^2^ carbons in GNFs (Figure S11A and Table S5, Supporting Information). This aligns with Raman spectroscopy, as evidenced by the higher intensity of the G peak compared to the D peak (Figure 3D). In the high‐resolution F 1s spectrum, a decrease in CF_3_ bonds was confirmed, accompanied by an increased area percentage of semi‐ionic C─F bonds and a slight decrease in covalent C─F bond percentage (Figure S11B and Table S5, Supporting Information). Fluorinating graphene commonly involves two competing reactions: Covalent C─F bond forming sp^3^‐hybridized C atoms connected to F atoms, and semi‐ionic C─F bonds, sp^2^ carbon associated with F atoms.^[^ ^38^ ^]^ This F 1s spectrum matched well with C 1s spectrum, indicating the successful GNFs formation after laser treatment. Our comprehensive molecular and chemical analyses, including FT‐IR and XPS measurements, demonstrate dynamic changes in GNF composition during laser graphitization. The observed increase in sp^2^ carbon formation underscored the enhanced properties of the materials after laser photothermal treatment. These findings offer valuable insights into the controlled transformation of the material and the influence of laser power on fluorine vaporization, sp^2^ carbon formation, and the evolution of C─F bonds, aligning closely with the observations from Raman spectroscopy analysis.

Thermal stability plays a crucial role in various applications like supercapacitors, batteries, electronics, composites, and coatings, preventing performance degradation, thermal runaway, and mechanical failure stemming from thermal changes experienced by graphene nanofibers. Our thermogravimetric analysis (TGA) on fPI NFs and GNFs produced under optimized laser conditions (Figure 3G) revealed a first‐stage decomposition over a broad range from room temperature to 486.97 °C, primarily associated with the evaporation of water molecules. However, in the second stage, fPI NFs experienced substantial loss of 91.31%, attributable to imide group structures, resulting in polymer chain rupture and the release of volatile compounds such as fluorine.^[^ ^39^ ^]^ In contrast, GNFs exhibited a weight loss of 35.74% in the second stage, which was primarily attributed to the non‐graphitized core of the nanofibers. This indicated that the core structure of GNFs retained partially polymeric characteristics, contributing to weight loss during thermal decomposition. Additionally, the carboxylic group decomposition and CO_2_ gas release also played a role in the observed weight loss (Figure S11, Supporting Information).^[^ ^40^ ^]^ This TGA demonstrates distinctive thermal behaviors between fPI NFs and GNFs, highlighting the superior thermal stability of GNFs, crucial for various practical applications involving graphene nanofibers.

### 2.3 Application of Laser‐Induced Graphene Nanofibers as Micro‐Supercapacitors for Next‐Generation Miniatured Energy Storage Devices

**Figure 4:**
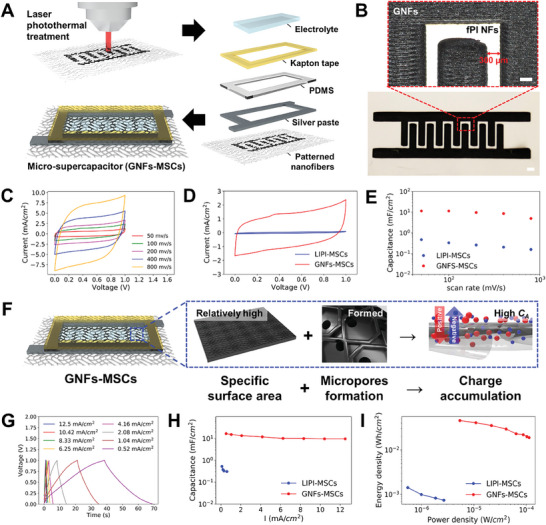
Electrochemical characterizations of GNFs‐MSCs and LIPI‐MSCs. A) Schematic illustration of the GNFs‐MSCs fabrication process. B) Digital photograph of GNFs‐MSCs featuring 12 interdigital electrodes with an optical microscope image depicting micro‐patterned GNFs (scale bar: 1 mm). C) CV curves of GNFs‐MSCs at a scan rate of 50 to 800 mV s^−1^. D) Cyclic voltammetry (CV) curves for GNFs‐MSCs and LIPI‐MSCs in 1 m H_2_SO_4_ at a scan rate of 100 mV s^−1^. E) Specific areal capacitance (*C_A_*) of GNFs‐MSCs and LIPI‐MSCs calculated from CV curves as a function of scan rate. F) Schematic illustration of higher charge accumulation through GNFs‐MSCs due to a larger surface area and microporous structure of GNFs. G) Galvanostatic charge‐discharge (CC) curves of GNFs‐MSCs at discharge current densities (*I_D_*) ranging from 0.5 to 12.5 mA cm^−2^. H) *C_A_* of GNFs‐MSCs and LIPI‐MSCs calculated from CC curves versus *I_D_*. I) Areal energy and power densities of GNFs‐MSCs and LIPI‐MSCs.

GNFs, characterized by high surface area, excellent electrical conductivity, superior electron mobility, and thermal stability, emerge as ideal candidates for energy storage applications, including supercapacitors and batteries. To demonstrate the potential of GNFs for energy storage applications, particularly leveraging their high specific surface area from meso‐ and micro‐porous structures, we developed GNFs‐based micro‐supercapacitors (GNFs‐MSCs) (**Figure** **4**A). Our approach involves crafting electrode patterns with GNFs, applying silver pasting on non‐patterned fPI NFs as interconnects, and using 1 m sulfuric acid (H_2_SO_4_) as an aqueous electrolyte. The laser‐induced transformation of fPI NFs then forms GNFs with a 300 µm interspacing (Figure 4B). The resulting micro‐supercapacitors exhibit exceptional attributes including high capacitance, high energy density, high power density, and remarkable cycling stability. The percolated network structure optimizes electrode material utilization by providing interconnected pathways for efficient ion and electron charge transport, large surface area for charge storage, and numerous electrochemically active sites for ion adsorption and desorption. This design, coupled with the flexibility of conductive nanofibers in flexible substrates, renders them suitable for flexible and wearable micro‐supercapacitor applications. The scalability of the percolated network structure supports large‐scale production, facilitating tailored micro‐supercapacitors with specific dimensions and performance characteristics, enhancing their mechanical stability and long‐term cycling performance within flexible and wearable applications.

To compare the performance of GNFs‐MSCs with laser‐induced polyimide film‐based micro‐supercapacitors (LIPI‐MSCs), we conducted cyclic voltammetry (CV) measurements, while the material characterizations of LIPI were reported in our prior works and referenced here.^[^ ^41^, ^42^ ^]^ Both GNFs‐MSC and LIPI‐MSCs displayed ideal electric double‐layer capacitor (EDLC) behavior, as evidenced by a pseudo‐rectangular shaped CV curve within the 0.0 to 1.0 V potential window at scan rates ranging from 50 to 800 mV s^−1^, highlighting its favorable characteristics for energy storage applications. The CV curves exhibited reversible or quasi‐reversible redox reactions at electrode‐electrolyte interface, primarily arising from the interaction between electrolyte ions and oxygen‐containing functional groups (such as C═O, ─COOH, and ─OH) present on the surface of GNFs. These functional groups actively participate in faradaic reactions during charge and discharge processes, contributing to pseud‐capacitance behavior and enhancing the charge storage capacity beyond that of a typical EDLC mechanism (Figure 4C; Figure S12A, Supporting Information).^[^ ^43^ ^]^ The hierarchical porous structure of GNFs facilitates the rapid diffusion of electrolyte ions to these active sites, supporting efficient redox reactions. Even at high scan rates, the reversibility of these reactions ensures reliable charge storage and release, resulting in high energy density and rapid charge/discharge rates, which are critical for applications in portable electronics and renewable energy systems. GNFs‐MSC exhibited a higher current density, indicating its potential for larger charge storage than LIPI‐MSCs (Figure 4D). The areal capacitance (*C_A_*) of GNFs‐MSCs, determined to be 11.41 mF cm^−2^ at a scan rate of 100 mV s^−1^, was over 33 times higher than that of LIPI‐MSCs (0.34 mF cm^−2^) and comparable to or exceeding that of EDLC‐type MSCs (Figure 4E; Table S7, Supporting Information).^[^ ^15^, ^44^ ^]^ The exceptional *C_A_* of GNFs‐MSCs is attributed to the extensive specific surface area of GNFs and the electrochemical activity enabled by their hierarchical porous structure (Figure 4F).^[^ ^45^ ^]^ The combined contributions of EDLC and pseudo‐capacitance mechanisms, driven by both physical adsorption and surface redox reactions, position GNFs‐MSCs as promising candidates for high‐performance energy storage applications.

To assess the power density and energy density of GNFs‐MSCs, galvanostatic charge–discharge (CC) measurements were conducted. The capacitive nature of GNFs‐MSCs was further confirmed by nearly triangular CC curves at various currents (0.52 to 12.5 mA cm^−2^) (Figure 4G), comparable to LIPI‐MSCs (Figure S13B, Supporting Information). GNFs‐MSCs exhibited significant *C_A_* of 16.8 mF cm^−2^ at a current density of 0.52 mA cm^−2^, surpassing reported EDLC‐type MSCs by over an order of magnitude (Table S8, Supporting Information).^[^ ^15^, ^44^, ^46^ ^]^ Furthermore, they retained 73% of their capacitance (9.7 mF cm^−2^) at a high current density of 12.5 mA cm^−2^, showcasing impressive rate‐retention capability (Figure 4H). This ability to maintain high capacitance at elevated current densities is advantageous for achieving high power densities, enhancing cycle life, and improving overall energy efficiency in GNFs‐MSCs. We assessed GNF‐based energy storage devices and LIPI‐MSCs using Ragone plots, comparing their energy and power density. Areal energy density (Wh cm^−2^) reflects stored energy per unit area, while areal power density (W cm^−2^) signifies the rate of energy transfer. Rightward points denote greater power density, indicating faster energy delivery or extraction, whereas higher points signify increased energy density, denoting enhanced energy storage per unit volume or mass. GNFs‐MSCs demonstrated nearly two orders of magnitude higher areal energy density (0.002 mW h cm^−2^) and areal power density (0.54 mW cm^−2^) than LIPI‐MSCs (Figure 4I). Furthermore, GNFs‐MSCs delivered more than two orders of magnitude higher energy density than that of EDLC‐type MSCs.^[^ ^15^, ^44^ ^]^ This exceptional performance is attributed to the distinctive fibrous and microporous structure of GNFs, providing abundant surface area for charge storage and efficient ion diffusion during charge–discharge cycles.

## 3 Conclusion

In summary, we presented a scalable and integrated approach for synthesizing graphene nanofibers (GNFs) by combining electrospinning with laser‐induced graphitization. This method enabled the efficient transformation of electrospun fluorinated polyimide nanofibers (fPI NFs) into GNFs with uniformly fibrous, meso‐ and microporous structures. The optimized laser photothermal treatment conditions (1.8 W, 3.5 in s^−1^, and 1000 PPI) facilitated key chemical and structural modifications, such as the formation of π‐bonded C═C networks (I_D_/I_G_ ratio of 0.856) and high‐quality graphene layers (I_2D_/I_G_ ratio of 0.911). The evaporation of fluorine during laser treatment further enhanced porosity and specific surface area, resulting in a robust framework suitable for energy storage applications. The GNFs demonstrated exceptional physical and electrochemical properties, including high thermal stability, conductivity, and performance in energy storage. GNFs‐based micro‐supercapacitors (GNFs‐MSCs) achieved remarkable areal capacitance (11.41 mF cm^−2^), representing a 33‐fold improvement compared to conventional LIPI‐MSCs, as well as significantly higher areal energy density (0.002 mW h cm^−2^) and areal power density (0.54 mW cm^−2^). These superior electrochemical characteristics highlight the critical role of the hierarchical porous structures of GNFs in optimizing electrolyte diffusion, charge transport, and charge storage. This study underscores the versatility and practicality of GNFs as next‐generation materials for micro‐sized energy storage devices (MESDs). The integrated fabrication approach not only ensures scalability but also positions GNFs as highly promising candidates for diverse applications, ranging from portable electronics to flexible and wearable devices. The unique combination of porosity, conductivity, and mechanical stability in GNFs provides innovative opportunities to address growing energy demands, paving the way for efficient and sustainable energy storage technologies.

## 4 Experimental Section

### Materials

4,4′‐(Hexafluoroisopropylidene)diphthalic anhydride (6FDA; C_19_H_6_F_6_O_6_, 99.0%) was purchased from Sigma–Aldrich Chemicals (MO, USA). 2,2′‐Bis(trifluoromethyl)benzidine (TFB; C_14_H_10_F_6_N_2_, >98.0%) was obtained from TCI chemicals (Japan). Finally, N,N‐Dimethylacetamide (DMA; C_4_H_9_NO, ≥99.5%) was purchased from Daejung Chemicals (Republic of Korea).

### Fabrication of Fluorinated Polyimide Nanofibers Through Electrospinning and Thermal Imidization

To prepare the precursor for fPI NFs, the fPAA solution was first prepared using the following steps: 0.1 mol of TFB was mixed in 32.6 mL DMA and stirred at room temperature under an N_2_ atmosphere until a homogeneous, colorless solution was formed. Then, 0.1 mol of 6FDA was added to the colorless solution in an ice bath, causing the solution to turn yellow. The mixture was stirred for 1 h under an N_2_ atmosphere, resulting in a highly viscous colorless solution. The mixture was vigorously stirred overnight at room temperature, and a 3.00 m fPAA solution was prepared for electrospinning. The homogeneous fPAA solution was used to synthesize fluorinated poly(amic)acid nanofibers (fPAA NFs) using horizontal electrospinning with a high‐voltage supply (Nano NC, Republic of Korea) and a syringe pump (Legato 100, KD Scientific, USA). The electrospun nanofibers were collected in an aluminum foil‐covered collector held 20.0 cm from the needle, under a flow rate of 0.5 mL h^−1^ and an applied voltage of 12.0 kV using 21‐gauge injection needles. The fabricated NFs were then dried at 70.0 °C in an oven overnight to remove any residual liquids. Finally, the fPAA NFs were thermally imidized in a vacuum oven at 250.0 °C for 3 h to form fPI NFs.

### Laser Graphitization of Polyimide Nanofibers

A 10.6 µm CO_2_ laser‐cutter system (VLS2.30, Universal Laser Systems, USA) was utilized to synthesize GNFs from the electrospun fPI NFs at laser power of 1.8 W, scan speed of 3.5 in s^−1^, and 1000 PPI. The pulse duration was fixed at ≈14 µs and the laser treatment was performed under ambient conditions.

### Characterization of Fabricated Nanofibers

The morphological characterization of GNFs was analyzed using an optical microscope (Cascade MPS150, Formfactor, USA), high‐resolution scanning electron microscopy (HR‐SEM; SU8010, Hitachi, Japan), and field‐emission transmission electron microscopy (FE‐TEM; JEM2100F, JEOL, Japan). The chemical structure and molecular interaction of the nanofibers were further analyzed using Raman spectroscopy (RAON‐Spec, NOST, Republic of Korea) and Fourier‐transform infrared spectroscopy (FT‐IR; JASCO FT‐IR 6600, Japan) in the range of 400–4000 cm^−1^. The crystalline structure of the nanofibers was analyzed using high‐resolution X‐ray diffraction (HR‐XRD; X'Pert‐PRO MRD, Philips, Netherlands), while X‐ray photoelectron spectroscopy (XPS) measurements were performed on a Thermo Fisher Scientific K‐alpha system (USA) to confirm the chemical composition and the chemical states of nanofibers. Finally, thermal stability was measured through thermogravimetric analysis (TGA; TG209F3, Netzsch, Germany).

### Electrochemical Characterization

Cyclic voltammetry and galvanostatic cyclic‐charge measurements were performed using a Gamry Reference 1010B potentiostat (USA) in a two‐electrode system. All measurements were conducted using aqueous electrolytes (1 M H_2_SO_4_) in ambient conditions. To ensure the full ions diffusion onto GNFs interface, the micro‐supercapacitors were soaked in electrolyte for 2 h before measurements. The areal capacitance (CA in mF cm^−2^) from the CV curves were calculated using Equation (1):^[^ ^15^, ^47^ ^]^

(1) $$C_{A}=\frac{1}{2\times A_{electrode}\times S\times \left(V_{f}-V_{i}\right)}\int_{V_{i}}^{V_{f}}I\left(V\right)dV$$

where *A_electrode_* is the surface area of active electrodes (in cm^2^) with 0.6 cm^2^ for the devices used in this work; *S* is the voltage sweep rate (in V s^−1^); *V_f_* and *V_i_* are the potential limits of CV curves; $\int_{V_{i}}^{V_{f}}I\left(V\right)dV$ is the integrated area from CV curves. The areal capacitance (*C_A_* in mF cm^−2^) based on CC curves were calculated using Equation (2):^[^ ^15^, ^47^ ^]^

(2) $$C_{A}=\frac{I}{A_{electrode}\times \left(\frac{\Delta V}{\Delta t}\right)}$$

where *I* is the discharge current (in amperes) and *ΔV/Δt* is the slope of the discharge curve. The areal energy density and areal power were quantified using Equations (3) and (4):^[^ ^15^, ^47^ ^]^

(3) $$E_{A}=\frac{1}{2}\times C_{A}\times \frac{{\left(\Delta V\right)}^{2}}{3600}$$

(4) $$P_{A}=\frac{E_{A}}{\Delta t}\;\;\times 3600$$

where *ΔV = V_max_−V_drop_* is the discharge potential range, *V_max_* is the maximum voltage (1 V), *V_drop_* is the voltage drop from the difference of the first two data points in the discharge curves, and *Δt* is discharge time (in s^−1^).

## Conflict of Interest

The authors declare no conflict of interest.

## Supporting information



Supporting Information

## Data Availability

The data that support the findings of this study are available from the corresponding author upon reasonable request.
