# Supplementary material for: Graphene Nanofibers by Integrated Manufacturing of Electrospinning and Laser Graphitization for Miniaturized Energy Storage Devices
Source: Adv Sci (Weinh). 2025 Mar 31;12(21):2414607. doi: 10.1002/advs.202414607 (PMC12140363; doi:10.1002/advs.202414607)
Supplement: Supplementary file 1 — Supporting Information [file ADVS-12-2414607-s001.docx]

Supporting Information

Graphene Nanofibers by Integrated Manufacturing of Electrospinning and Laser Graphitization for Miniaturized Energy Storage Devices

Bumjun Park^§^, Shirin Movaghgharnezhad^§^, Seung Min Lee, Yonghyeon Park, Sejin Son*, Yun Suk Huh*, Pilgyu Kang*

B. Park, Y. Park, S. Son, Y. S. Huh

Department of Biological Sciences and Bioengineering, Nano Bio High-Tech Materials Research Center, Inha University, Michuhol-gu, Incheon 22212, Republic of Korea
E-mail: yunsuk.huh@inha.ac.kr, ssejin@inha.ac.kr

S. Movaghgharnezhad, S. M. Lee, P. Kang

Department of Mechanical Engineering, George Mason University, Fairfax, Virginia 22030, USA

E-mail: pkang7@gmu.edu

P. Kang

Quantum Science and Engineering Center, George Mason University, Fairfax, Virginia 22030, USA

E-mail: pkang7@gmu.edu

§ These authors contributed equally to this work.


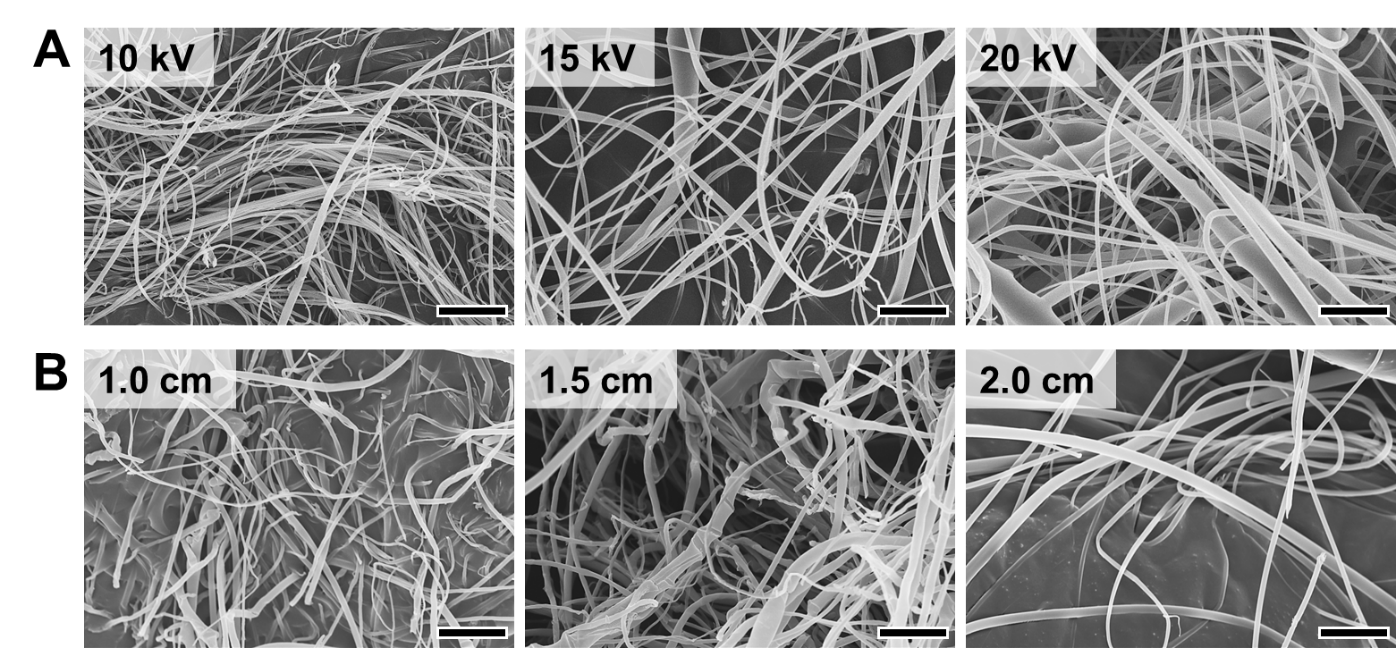


**Figure S1.** The optimal SEM images of fabricated fPAA NFs at different **A** applied voltages, and **B** distance between needle and collector (DNC). All scale bars indicate 10.0 μm.


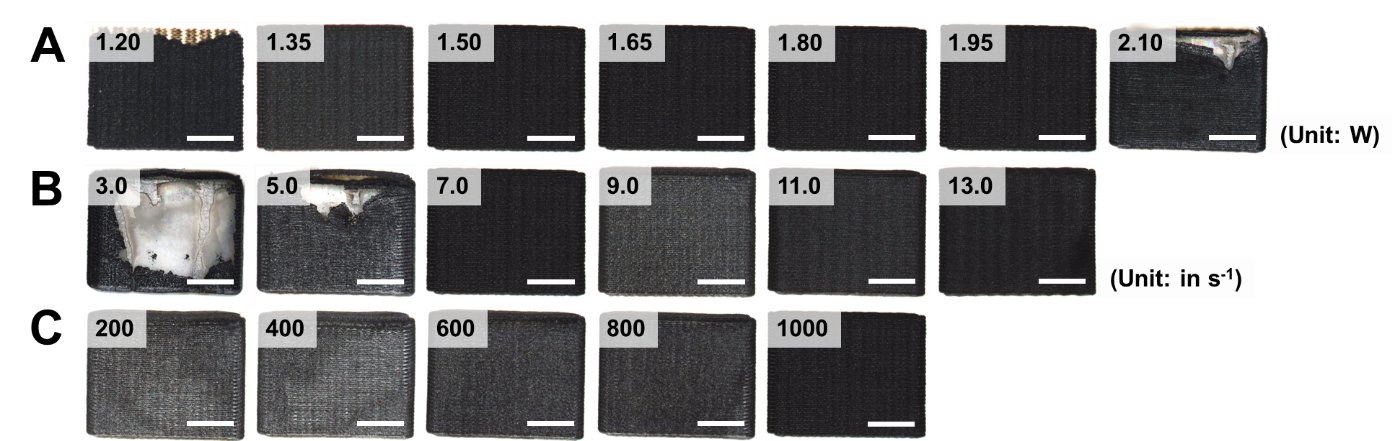


**Figure S2.** The optical microscope image of fabricated GNFs at different **A** laser powers, **B** speeds, and **C** PPI. All scale bars indicate 1.0 mm.


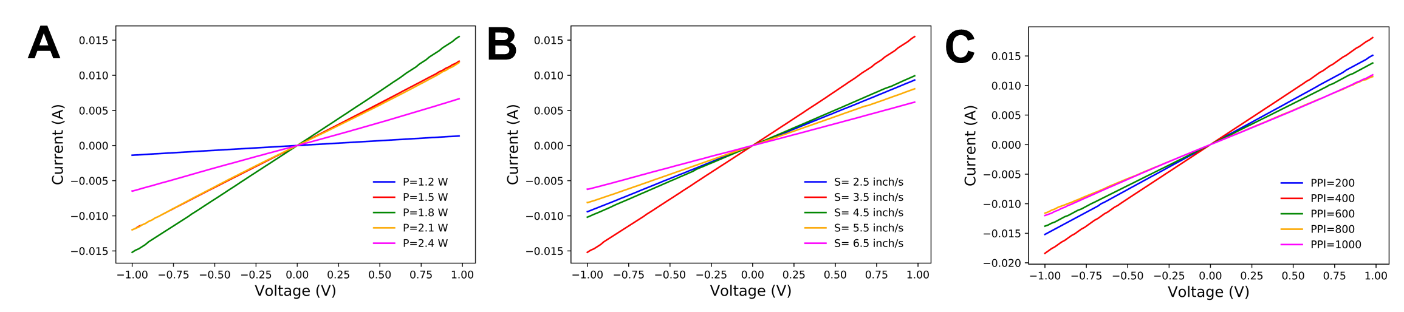


**Figure S3.** I-V measurement graphs of fabricated GNFs at different **A** laser powers, **B** speeds, and **C** PPI.


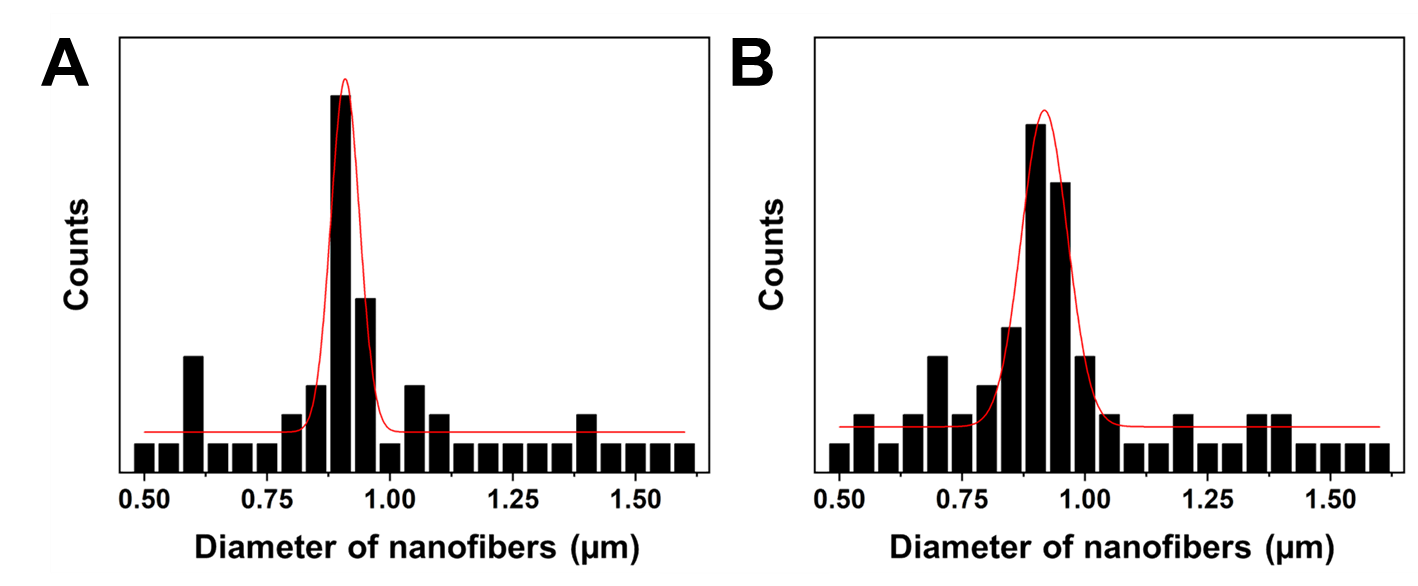


**Figure S4.** Histogram of the diameter of fabricated **A** fPI NFs after two-step synthesis procedure of electrospinning and thermal imidization and **B** GNFs.


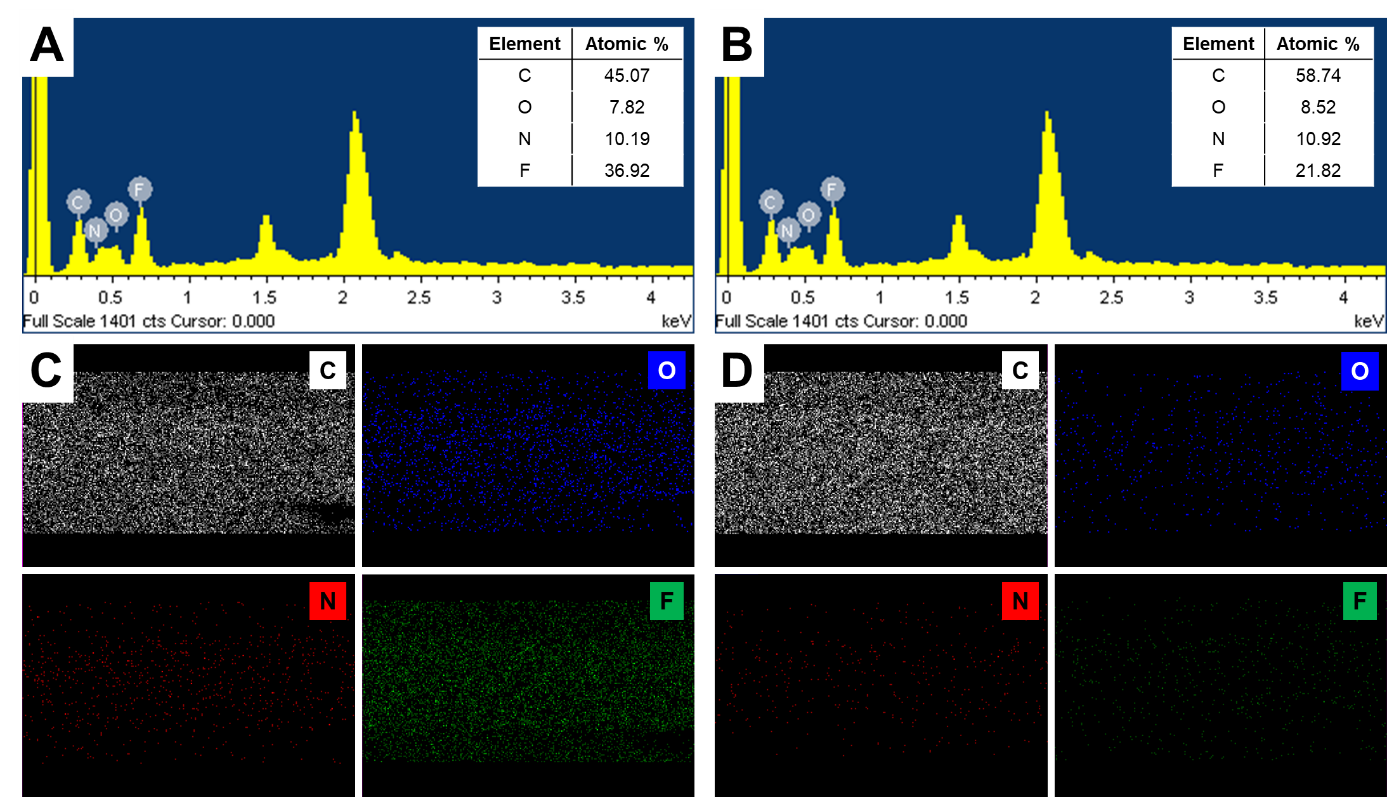


**Figure S5.** EDS spectrum of **A** fPI NFs and **B** GNFs. Elemental mapping images obtained from **C** fPI NFs and **D** GNFs for elements C, O, N and F, respectively.


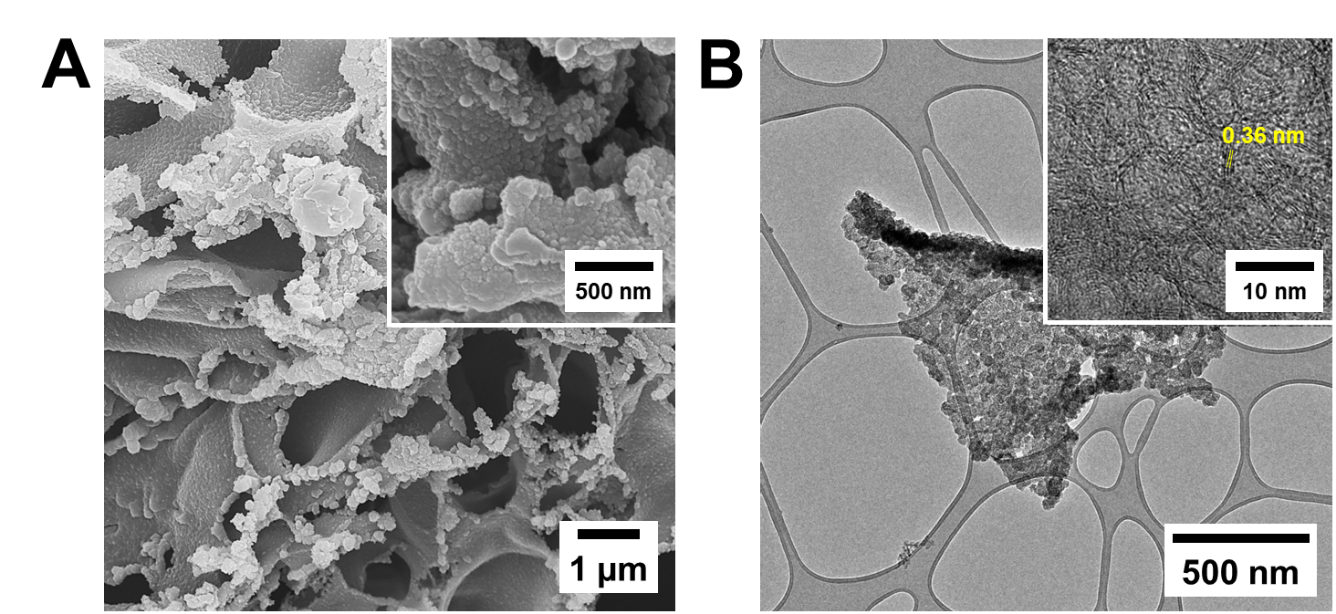


**Figure S6.** **A** SEM and **B** TEM images of GNFs at laser power of 2.1 W.


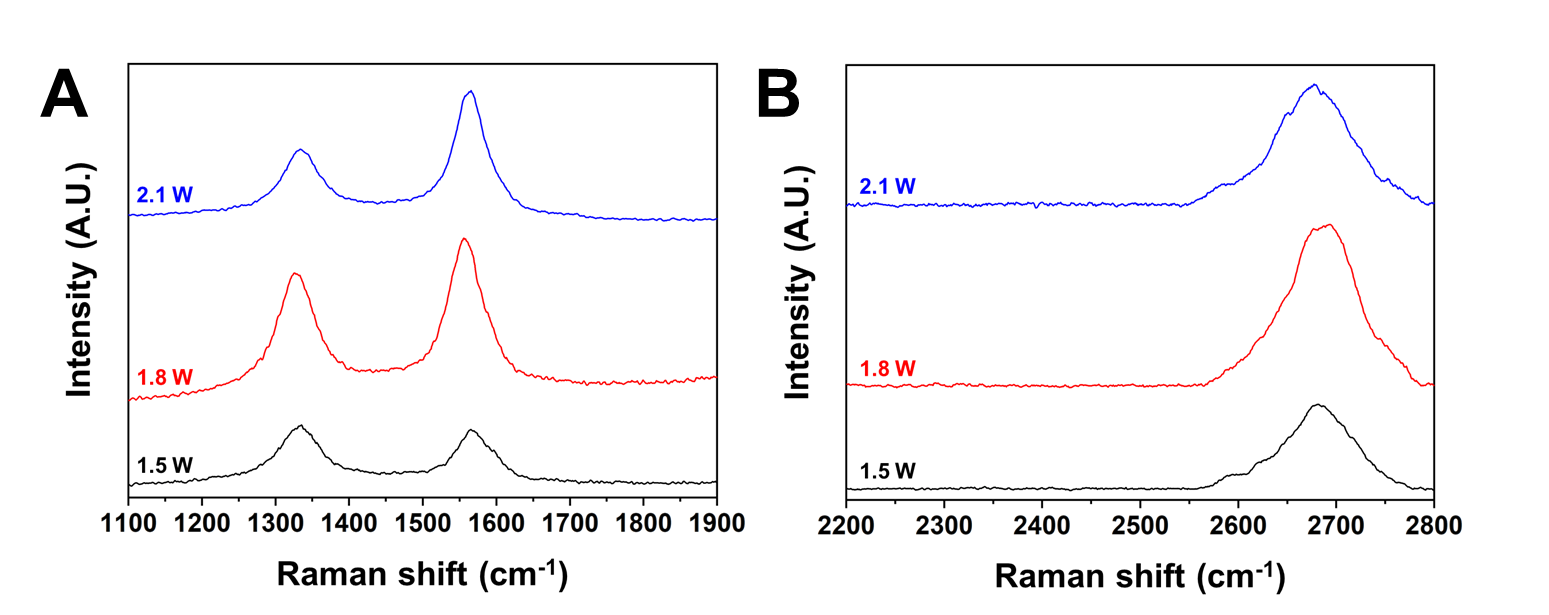


**Figure S7.** Raman spectroscopy analysis of GNFs at the range of **A** 1100 to 1900 cm^-1^ and **B** 2200 to 2800 cm^-1^ under various power from 1.5 W to 2.1 W.


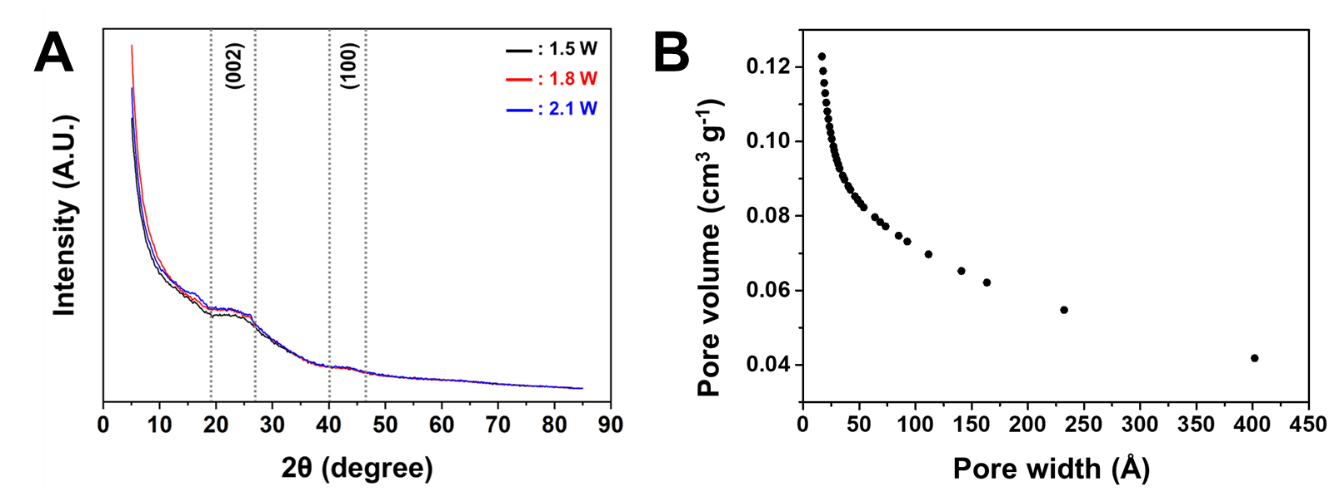


**Figure S8.** **A.** XRD spectra of GNFs under various power from 1.5 W to 2.1 W, and **B.** BET pore size distribution for GNFs.


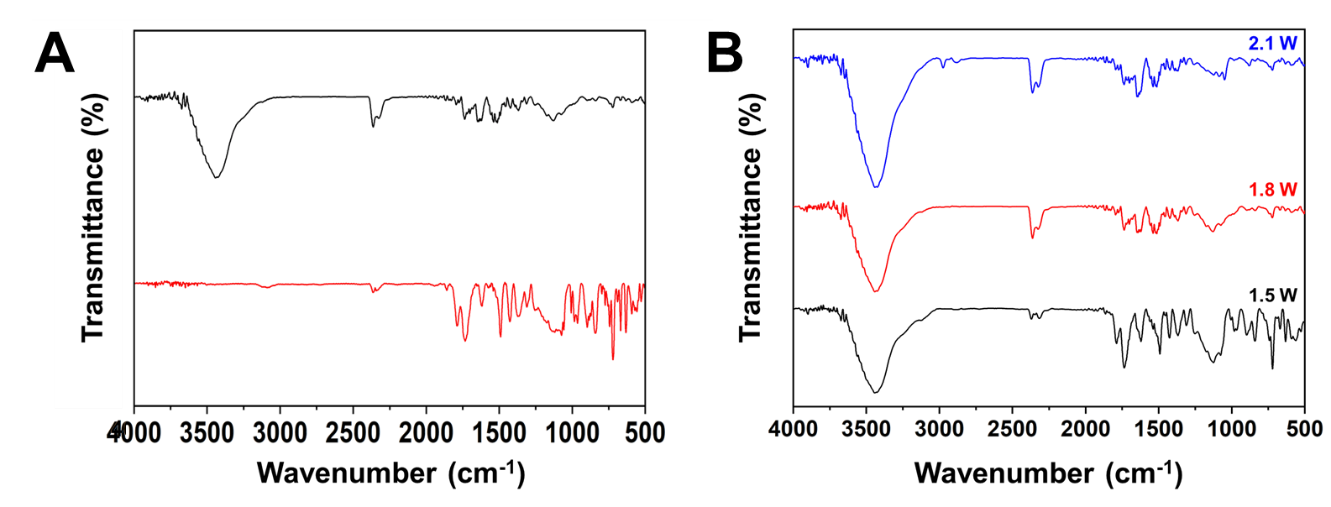


**Figure S9. A.** FT-IR spectra of fabricated NFs, in which the black and red curves indicate GNFs and fPI NFs, respectively, and **B** FT-IR spectra of GNFs under various power from 1.5 W to 2.1 W.


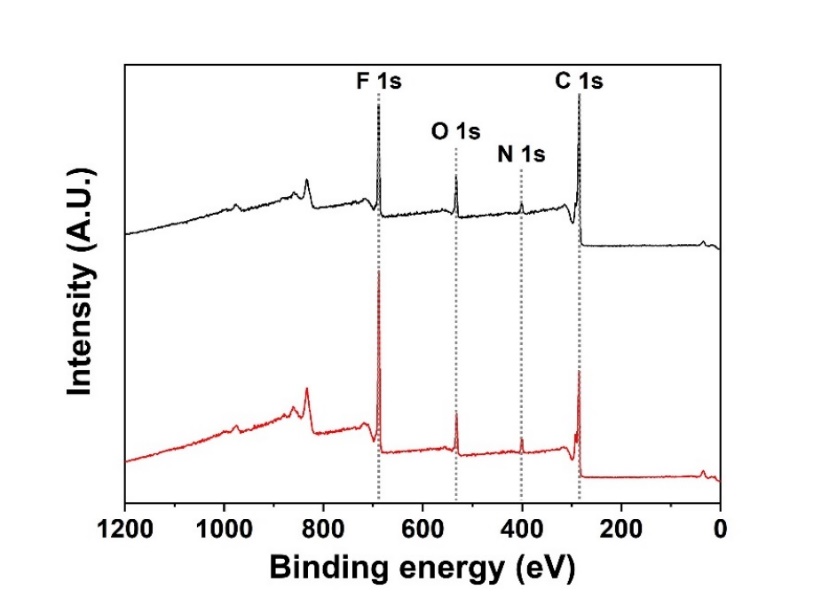


**Figure S10.** Wide scan XPS spectra of fabricated nanofibers. The black and red line indicates GNFs and fPI NFs, respectively.


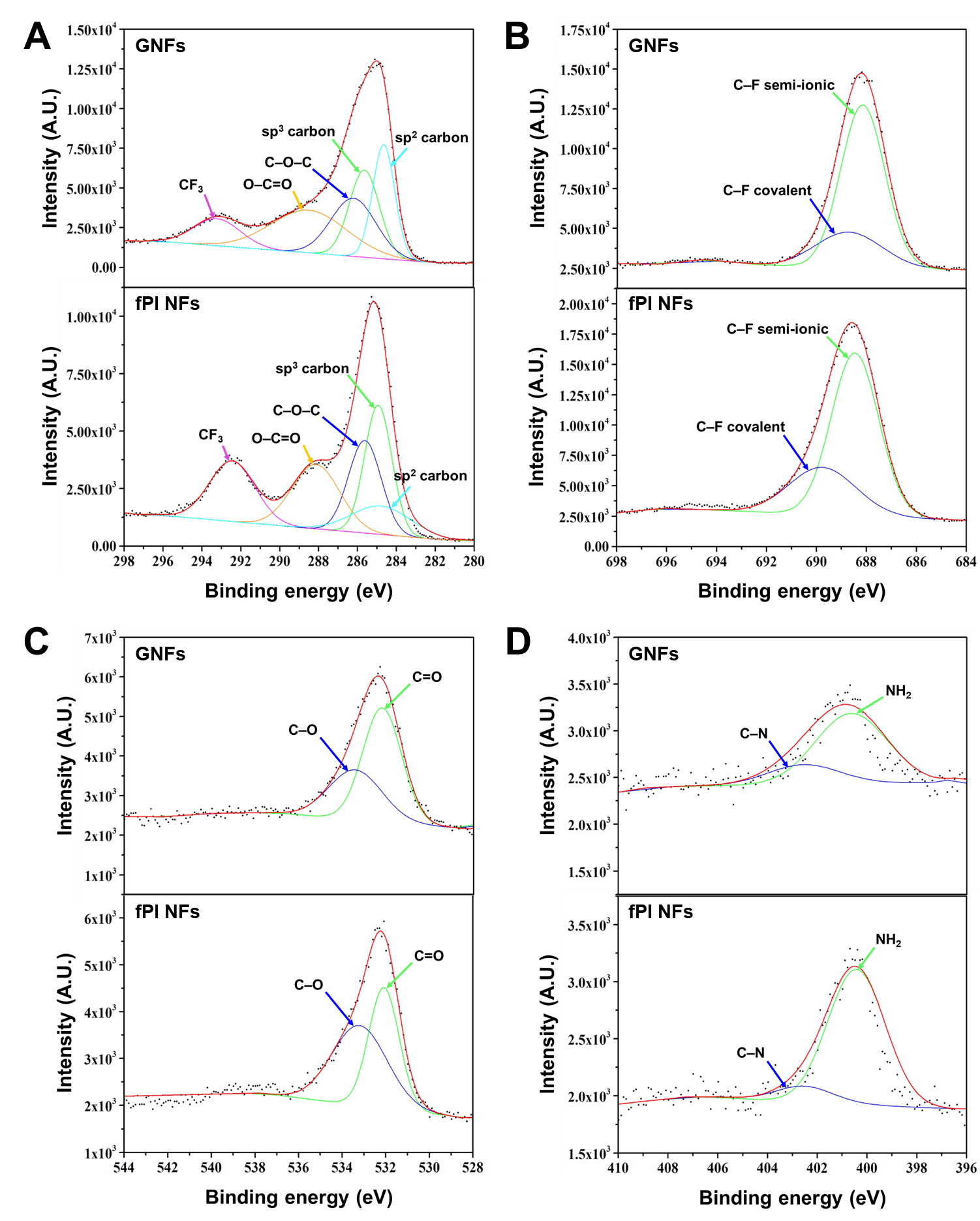


**Figure S11.** Narrow scans of **A** C 1s, **B** F 1s, **C** O 1s and **D** N 1s for GNFs and fPI NFs.


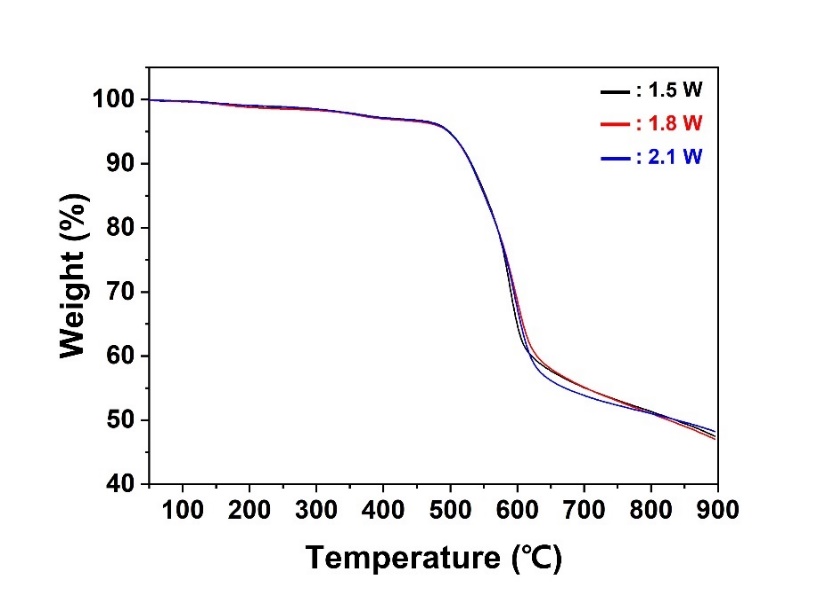


**Figure S12.** TGA curves of GNFs under various power from 1.5 W to 2.1 W.


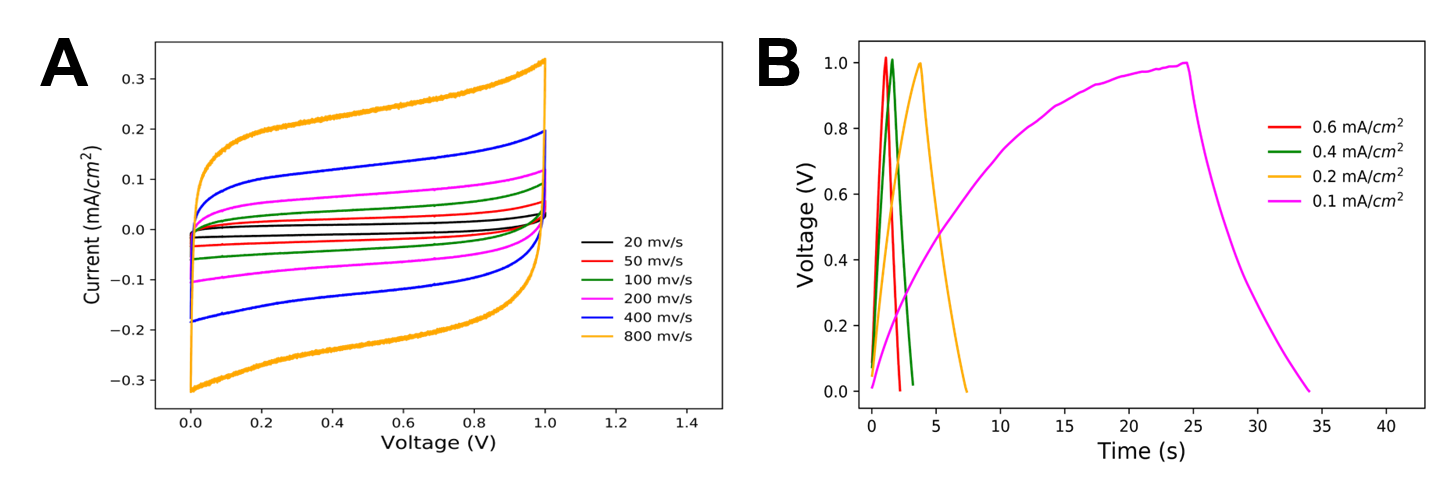


**Figure S13.** **A** CV curves of LIPI-MSCs at scan rates from 20 to 800 mV s^-1^. **B** Galvanostatic CC curve of LIPI-MSCs at current densities from 0.1 to 0.6 mA cm^-2^.


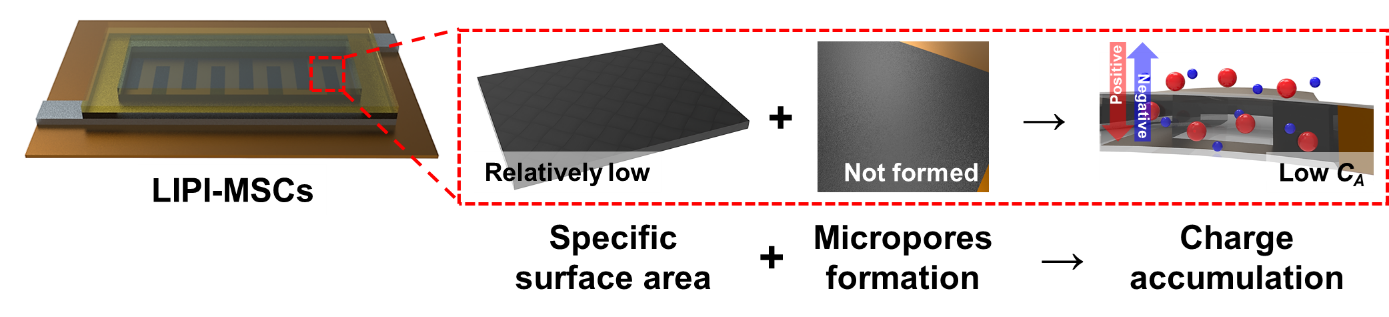


**Figure S14.** Schematic illustration of charge accumulation through LIPI-MSCs.

**Table S1.** Specific peak values of fPI NFs analyzed through Raman spectroscopy.

| **Peak (cm^-1^)** | **Allocation** | **Peak (cm^-1^)** | **Allocation** |
| --- | --- | --- | --- |
| 738.91 | C−F vibration | 1123.55 | C−N−C stretching |
| 1154.95 | Benzene ring | 1232.42 | CH deform |
| 1304.44 | CH_2_ twist | 1379.18 | Axial C−N−C stretching |
| 1435.49 | CH_2_ bend | 1620.14 | Benzene ring |
| 1786.35 | C=O stretching |  | |

**Table S2.** I_D_/I_G_ ratio of GNFs under various power of laser.

| **Laser power (W)** | **D peak intensity** | **G peak intensity** | **I_D_/I_G_** |
| --- | --- | --- | --- |
| 1.5 | 2378.61 | 2322.54 | 1.024 |
| 1.8 | 2531.21 | 2956.88 | 0.856 |
| 2.1 | 2260.92 | 2969.54 | 0.761 |

**Table S3.** FWHM value and I_2D_/I_G_ ratio of GNFs under various power of laser.

| **Laser power (W)** | **FWHM value** | **2D peak intensity** | **I_2D_/I_G_** |
| --- | --- | --- | --- |
| 1.5 | 80.21 | 2681.23 | 1.154 |
| 1.8 | 81.35 | 2692.03 | 0.911 |
| 2.1 | 86.27 | 2677.19 | 0.902 |

**Table S4.** Specific peak values of fPI NFs and GNFs analyzed through FT-IR.

| **fPI NFs** | | **GNFs** | |
| --- | --- | --- | --- |
| **Peak (cm^-1^)** | **Allocation** | **Peak (cm^-1^)** | **Allocation** |
| 500.00 – 1000.00 | C=C bending | 500.00 – 1000.00 | C=C bending |
| 1312.29, 1369.03 | C−F bond | 1311.94, 1373.13 | C−F bond |
| 1619.88 | C=C stretching | 1619.88 | C=C stretching |
| 1731.87, 1788.61 | C=O bond | 1735.82 | C=O bond |
| 1857.29, 1942.41 | C−H bond |  | |

**Table S5.** Atomic compositions and atomic ratio of fPI NFs and GNFs.

| **Element** | **Atomic percentage (%)** | |
| --- | --- | --- |
|  | **fPI NFs** | **GNFs** |
| C 1s | 63.02 | 74.46 |
| O 1s | 7.36 | 6.85 |
| N 1s | 4.15 | 2.82 |
| F 1s | 25.48 | 15.88 |

**Table S6.** Specific peak values and area percentage of fPI NFs and GNFs under high resolution XPS spectra.

| **Atom** | **Binding energy**  **(eV)** | **Chemical composition** | **Area percentage (%)** | |
| --- | --- | --- | --- | --- |
|  |  |  | **fPI NFs** | **GNFs** |
| C 1s | 284.79 | sp^2^ carbon | 12.05 | 25.62 |
|  | 284.93 | sp^3^ carbon | 25.01 | 21.30 |
|  | 285.63 | C−O−C | 21.51 | 21.30 |
|  | 288.14 | O−C=O | 21.92 | 21.49 |
|  | 292.43 | CF_3_ | 19.51 | 10.29 |
| F 1s | 688.46 | semi-ionic C−F | 71.04 | 75.75 |
|  | 689.77 | covalent C−F | 28.96 | 24.25 |
| O 1s | 532.08 | C=O | 45.61 | 42.66 |
|  | 533.20 | C−O | 54.39 | 57.34 |
| N 1s | 400.43 | NH_2_ | 89.46 | 88.08 |
|  | 400.52 | C−N | 10.54 | 11.92 |

**Table S7.** Areal capacitance values of two different micro-supercapacitors under various scan rates.

| **Scan rate (mV s^-1^)** | **Areal capacitance value (mF cm^-2^)** | |
| --- | --- | --- |
|  | **LIPI-MSCs** | **LIGF-MSCs** |
| 50 | 0.48 | 11.44 |
| 100 | 0.34 | 11.41 |
| 200 | 0.26 | 9.63 |
| 400 | 0.21 | 8.59 |
| 800 | 0.16 | 4.98 |

**Table S8.** Comparison of the electrochemical performance of graphene nanofibers-based micro-supercapacitor with other devices reported in the literature.

|  | **Areal capacitance**  **(mF cm^-2^)** | **Energy density**  **(mWh cm^-2^)** | **Power density**  **(mW cm^-2^)** |
| --- | --- | --- | --- |
| Laser-induced graphene^[1]^ | ~4 | ~0.0001 | ~9 |
| Graphene oxide (GO)^[2]^ | ~0.081 | ~NA | ~NA |
| Laser-induced graphene oxide^[3]^ | ~0.51 | ~NA | ~NA |
| Laser-scribed graphene oxide^[4]^ | ~2.32 | ~NA | ~NA |
| 3D printed Carbon Nanotube^[5]^ | ~0.39 | NA | NA |
| Laser-induced MOF derived graphene^[6]^ | 1.36 | 0.14 | 580 |
| Graphene nanofibers  (This study) | 11.41-16.80 | 0.002 | 0.54 |

**References**

[1] J. Lin, Z. Peng, Y. Liu, F. Ruiz-Zepeda, R. Ye, E. L. Samuel, M. J. Yacaman, B. I. Yakobson, J. M. Tour, *Nat. Commun.* **2014**, 5, 5714.

[2] M. F. El-Kady, V. Strong, S. Dubin, R. B. Kaner, *Science* **2012**, 335, 1326.

[3] Wu, Z. S., Parvez, K., Feng, X. L. & Mullen, K. , *Nat. Commun.* **2013**, 4, 2487.

[4] W. Gao, N. Singh, L. Song, Z. Liu, A. L. M. Reddy, L. Ci, R. Vajtai, Q. Zhang, B. Wei, P. M. Ajayan, *Nat. Nanotechnol.* **2011**, 6, 496; d) M. F. El-Kady, R. B. Kaner, *Nat. Commun.* **2013**, 4, 1475.

[5] W. Yu, H. Zhou, B. Q. Li, S. Ding, *ACS Appl. Mater. Interfaces* **2017**, 9, 4597

[6] A. Basu, K. Roy, N. Sharma, S. Nandi, R. Vaidhyanathan, S. Rane, C. Rode, S. Ogale, *ACS Appl. Mater. Interfaces* **2016**, 8, 31841.
